# Plasma DNA concentration as a predictor of mortality and sepsis in critically ill patients

**DOI:** 10.1186/cc4894

**Published:** 2006-04-13

**Authors:** Andrew Rhodes, Stephen J Wort, Helen Thomas, Paul Collinson, E David Bennett

**Affiliations:** 1 Intensive Care Unit, St Georges's Hospital, London, UK; 2 Department of Chemical Pathology, St Georges's Hospital, London, UK

## Abstract

**Introduction:**

Risk stratification of severely ill patients remains problematic, resulting in increased interest in potential circulating markers, such as cytokines, procalcitonin and brain natriuretic peptide. Recent reports have indicated the usefulness of plasma DNA as a prognostic marker in various disease states such as trauma, myocardial infarction and stroke. The present study assesses the significance of raised levels of plasma DNA on admission to the intensive care unit (ICU) in terms of its ability to predict disease severity or prognosis.

**Methods:**

Fifty-two consecutive patients were studied in a general ICU. Blood samples were taken on admission and were stored for further analysis. Plasma DNA levels were estimated by a PCR method using primers for the human β-haemoglobin gene.

**Results:**

Sixteen of the 52 patients investigated died within 3 months of sampling. Nineteen of the 52 patients developed either severe sepsis or septic shock. Plasma DNA was higher in ICU patients than in healthy controls and was also higher in patients who developed sepsis (192 (65–362) ng/ml versus 74 (46–156) ng/ml, *P *= 0.03) or who subsequently died either in the ICU (321 (185–430) ng/ml versus 71 (46–113) ng/ml, *P *< 0.001) or in hospital (260 (151–380) ng/ml versus 68 (47–103) ng/ml, *P *< 0.001). Plasma DNA concentrations were found to be significantly higher in patients who died in the ICU. Multiple logistic regression analysis determined plasma DNA to be an independent predictor of mortality (odds ratio, 1.002 (95% confidence interval, 1.0–1.004), *P *= 0.05). Plasma DNA had a sensitivity of 92% and a specificity of 80% when a concentration higher than 127 ng/ml was taken as a predictor for death on the ICU.

**Conclusion:**

Plasma DNA may be a useful prognostic marker of mortality and sepsis in intensive care patients.

## Introduction

Prognosis of patients is important in risk stratification and for efficient use of hospital resources. Predicting the outcome of patients in the intensive care environment is of particular significance, to ensure that resources are used appropriately. Numerous biomarkers have been evaluated to predict morbidity and mortality in the intensive care setting, although none have proved entirely useful. Examples of such biomarkers include cytokines [1], procalcitonin [2], C-reactive protein [3], brain natriuretic peptide [4] and cardiac troponin I [5].

Interest has recently developed in the use of plasma DNA, or cell-free nucleic acid, as a prognostic marker [6]. Plasma DNA can be defined as fragments of DNA that are detectable in the extracellular fluid. There are two types of DNA present in the circulation; 'free' DNA present in the plasma (which includes DNA packed into nucleosomes from apoptotic cells) or DNA associated with circulating lymphocytes (considered a minor component) [7].

Relatively little is known about free circulating DNA, but numerous studies have established that baseline levels are present in normal, healthy populations, albeit at very low levels [8]. It has been suggested that DNA enters the circulation following cell death. This can be as a result of cell necrosis or of programmed cell death (apoptosis). In addition, the clearance mechanism of DNA from the circulation is poorly understood, although experimental studies using animals have produced evidence suggesting that the liver and the kidneys are prime candidates for its removal [9]. An increase in plasma DNA concentration may therefore occur either due to increased liberation from cells or to a decrease in clearance efficiency.

Increased plasma levels of DNA have been found in conditions associated with cell death, including cancer [8], pregnancy [10], stroke [6], myocardial infarction [11] and trauma [12]. In addition, the plasma DNA concentration in trauma patients is directly related to the severity of injury [12]. Furthermore, the plasma DNA concentration taken soon after the accident is predictive of death with a sensitivity and a specificity of 100% and 81%, respectively [12]. A prognostic value has also been shown in patients with acute myocardial infarction [11] and acute stroke [6].

Sepsis is a major cause of morbidity and mortality in patients in the ICU [13]. Sepsis is associated with cell necrosis and apoptosis [14]. Indeed, plasma DNA levels have been shown to be increased in patients with sepsis [15]. Furthermore, elevated nucleosome levels, a marker of cell apoptosis, are increased in patients with severe sepsis and septic shock [16]. The aim of the present study was to investigate the prognostic value of circulating levels of cell-free DNA in patients with sepsis in the intensive care setting.

## Materials and methods

Unselected, consecutive admissions to St George's Hospital General Intensive Care Unit (ICU) were prospectively entered into this study. The General ICU at St George's Hospital caters for general medical and general surgical patients with no cardiac or neurosurgical specialties. Informed consent from the patients was waived by the local research/ethics committee as this was an observational study looking at plasma DNA, a new variable measured from blood samples that were taken as part of standard clinical practice. All blood samples were taken from indwelling intra-arterial access on admission to the ICU.

Demographic and medical data were collected that included details on the reason for admission, complications in the first 24 hours, the length of stay, ICU and hospital mortality, and the Sepsis-related Organ Failure Assessment (SOFA) score [17]. Patients were followed up three months after discharge from the ICU. Severe sepsis and septic shock were defined according to previously published criteria [18].

Ethylenediamine tetraacetic acid samples were collected from each patient. All samples were separated within 3 hours. In addition, samples were also taken from 10 healthy volunteers to determine the plasma DNA concentrations in a healthy population. To ensure cell-free plasma collection, samples were initially centrifuged for 6 minutes at 3,000 rpm, followed by separation into a 1.5 ml clear polypropylene tube taking care not to disturb the buffy coat layer. The newly separated aliquot was centrifuged for a further 10 minutes at 14,000 rpm, after which the upper portion of plasma was removed by a pasteur pipette (approximately 500 μl), was placed into a further clear tube and was frozen at -20°C prior to extraction.

Extraction of DNA from 134 plasma samples was accomplished using the High Pure PCR Template Preparation kit (Roche, Lewes, UK). The only adaptation made to the manufacturer's protocol was the use of 200 μl plasma rather than whole blood. Plasma DNA was detected by quantitative PCR (Roche Lightcycler; Roche, Lewes, UK), using primers for the β-globin gene, a housekeeping gene involved in the formation of a functioning haemoglobin molecule. This gene has been used successfully in previous studies measuring plasma DNA using similar PCR-based techniques [12]. The generation of a plasma DNA standard curve was accomplished using human genomic DNA.

The 101-base-pair amplicon was detected using primer sequences and were verified in the Genbank database (accession number U01317) [12]. The primer sequence and concentration were obtained such that a concentration of 300 nmol/l was present in the final mixture [12]. The annealing temperature of the primers was set at 60°C. The optimal magnesium chloride (MgCl_2_) concentration was determined by assessing at which concentration the lowest crossing point, the highest fluorescence intensity and the steepest curve slope were observed. Absolute quantification of plasma DNA was achieved using the Lightcycler software (version 3.5.2; Roche, Lewes, UK). On each run, a standard curve was repeated in duplicate as well as with the inclusion of a 'positive' genomic DNA control and a negative (de-ionised water) control.

### Statistical analyses

Data are presented either as absolute numbers, as medians with the interquartile range or as percentages. Statistical significance was set at a level of *P *= 0.05. Statistical variance between groups was assessed by the Fisher's exact test for discrete variables and by the Mann-Whitney *U* test for continuous variables. Spearman-rank correlation was used for all correlation data. A backwards logistic regression model was set up to identify factors that had independent predictive value for mortality on the ICU. All the factors (described later) were entered into the model. The least significant factor was then removed from the model and the calculation repeated until all remaining factors remained significant. Receiver operator characteristic (ROC) curve analysis was used to estimate an optimal cutoff value for the use of plasma DNA measurements for predicting death as well as the sensitivity and specificity of the test at this level.

## Results

**Figure 1:**
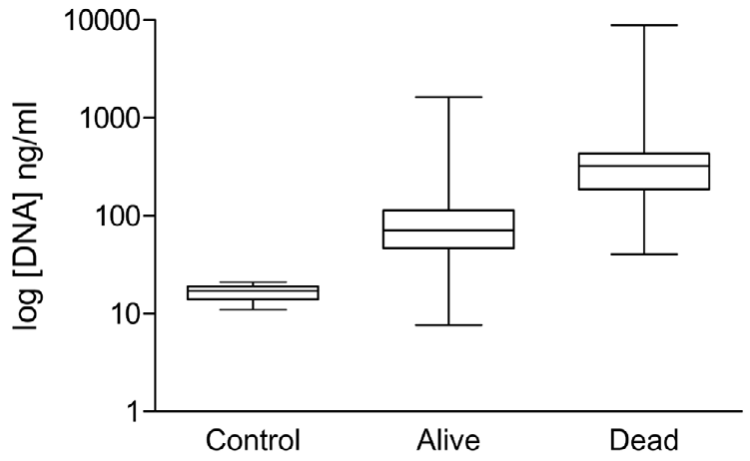
Box and whisker plot for plasma DNA levels between controls and patients who survived or died in the intensive care unit. The box represents a median and interquartile range, whereas the whiskers represent the range.

Fifty-two patients were recruited into the study. Baseline characteristics of these patients are presented in Table 1. The median SOFA score on admission to the ICU was 5.5 (3–8). Nineteen of the 52 (37%) patients had a diagnosis of either sepsis or septic shock within the first 24 hours of admission. Thirteen of the 52 (25%) patients died in the ICU and 16 patients (31%) died in hospital. The median length of stay of patients on the ICU was 5 (2–12) days and the median length of stay in hospital was 14 (7–30) days. The 10 healthy controls had a median plasma DNA level of 17 (14–19) ng/ml. The median plasma DNA level for all patients on admission to the ICU was significantly higher than that of the controls (80 (48–260) ng/ml, *P *= 0.001) (Figure 1).

### Plasma DNA and severity of illness

There were no differences seen in the plasma DNA between patients who did or did not require mechanical ventilation within the first 24 hours (*P *= 0.27) or between patients who were operated on or not (*P *= 0.26). Plasma DNA was higher in patients who, within the first 24 hours, required renal support (224 (75–429) ng/ml versus 79 (47–239) ng/ml, *P *= 0.07) or inotropic support (246 (74–424) ng/ml versus 70 (46–153) ng/ml, *P *= 0.007). There was a significant correlation between the plasma DNA and the SOFA score (*r*^2 ^= 0.2, *P *= 0.002).

### Plasma DNA and outcome

Patients with a diagnosis of severe sepsis or septic shock had higher levels of plasma DNA than those without the diagnosis (192 (65–362) ng/ml versus 74 (46–156) ng/ml, *P *= 0.03). No correlation was found between plasma DNA and C-reactive protein, N Terminal-brain natriuretic peptide or procalcitonin. Plasma DNA was significantly higher in patients who died in intensive care or in the hospital following discharge from the ICU (Table 2). The plasma DNA level was significantly correlated with the length of stay on the ICU for survivors (*r*^2 ^= 0.12, *P *= 0.03) but not with the length of stay in hospital. Sequential DNA levels were measured for 20 patients. Although there was a trend to persistently higher plasma DNA levels in patients who died or developed sepsis compared with those who did not develop sepsis or survived, this difference did not reach statistical significance (data not shown).

### Prognostic ability of plasma DNA

**Figure 2:**
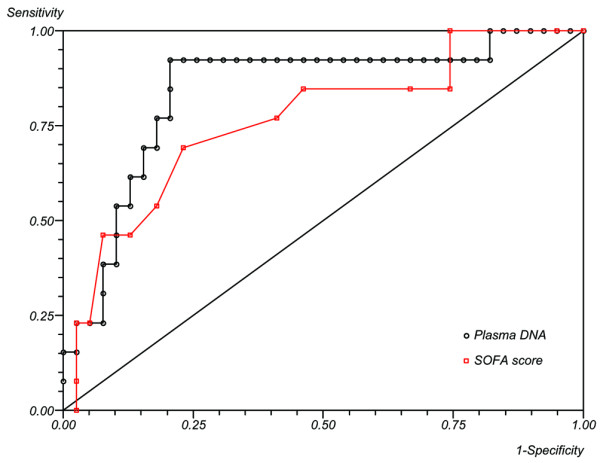
Receiver operating characteristic curves for plasma DNA and the Sepsis-related Organ Failure Assessment (SOFA) score to predict intensive care outcome. The area under the curve for plasma DNA is 0.84 (95% confidence interval, 0.71–0.97) and that for the SOFA score is 0.76 (95% confidence interval, 0.61–0.92).

ROC curves were calculated for the use of plasma DNA as a predictor of either ICU death or hospital death and for the SOFA score to predict ICU mortality (Figure 2). The area under the ROC curves for plasma DNA to predict ICU and hospital deaths were 0.84 (95% confidence interval, 0.71–0.97) and 0.79 (95% confidence interval, 0.63–0.94). The area under the ROC curve for the SOFA score to predict intensive care outcome was 0.76 (95% confidence interval, 0.61–0.92). The optimal cutoff value for plasma DNA to predict ICU mortality and hospital mortality was 127 ng/ml. This cutoff value gave a sensitivity of 92% and a specificity of 80% for ICU mortality and a sensitivity of 81% and a specificity of 81% for hospital mortality.

A univariate analysis was performed to compare various factors as predictors of mortality (Table 3). At intensive care admission, the SOFA score and the plasma DNA concentration were the only significant variables at predicting outcome. Using a backwards logistic regression model, plasma DNA was the only independent factor that could predict ICU mortality (odds ratio, 1.002 (95% confidence interval, 1.0–1.004), *P *= 0.05).

## Discussion

In the present study plasma DNA levels were found to be significantly higher in patients who did not survive hospital admission than in patients who survived. In addition, plasma DNA levels were significantly higher in those patients who did not survive the ICU stay than in those that were discharged to general wards. Indeed, when used as a predictor of ICU survival, the sensitivity and specificity of the plasma-free DNA concentration as a predictor of survival were 92% and 80%, respectively, with a likelihood ratio of 10.3, superior to that of the SOFA score. Furthermore, plasma DNA levels were significantly higher in those patients that developed sepsis compared with in patients that did not. The results presented here clearly demonstrate that plasma DNA may be a useful prognostic marker of mortality and sepsis in intensive care patients. Although a recent study has demonstrated that plasma DNA levels were higher in patients that died in the ICU (from all causes) [19], our study extends this finding to hospital mortality at three months. Furthermore, although a recent study demonstrated raised nucleosome-associated DNA in the plasma of patients with severe sepsis and septic shock [16], our study is the first to demonstrate the utility of 'total' circulating plasma DNA as a predictor of sepsis in the ICU setting.

Plasma DNA concentrations measured in this study are consistent with those previously reported [12,20]. In particular, in an ICU population post trauma, patients with mild/moderate trauma had circulating DNA values close to the septic patients described in our study. Patients with severe trauma have been shown to have much higher values [12]. A recent study attempting to determine a 'normal reference range' in Taiwanese medical students revealed a mean of 57.1 ng/ml and an upper cutoff limit of 118 ng/ml [8]. If this value was used in the present study, approximately one-half of the ICU patients would be within the normal range. However, we feel this difference highlights the dependence of results upon assay methodology (such as, different PCR-based methodology), highlights a possible racial variation and highlights the need for a common reference range. Finally, we must state that our healthy control group was not age matched with the study population. Although age itself may theoretically be a determinant of plasma DNA concentration, we could not find any actual evidence for this in similar studies. In addition, our control values correspond well with other control groups used in such studies.

Measuring circulating DNA has been shown to be useful for early risk stratification and prediction of inhospital and overall morbidity and mortality in a range of conditions including stroke [6], myocardial infarction [11], cancer [8] and trauma [12]. In stroke patients, the plasma DNA concentration is better at predicting mortality and 6-month morbidity after acute stroke than a combination of neuroimaging techniques and clinical assessment. Plasma DNA could not, however, differentiate between cerebral infarction and haemorrhage, suggesting that a wide range of insults may increase levels of circulating DNA. It appears that circulating DNA in patients with cancer is almost certainly derived from tumour cells, as specific genetic alterations are common to both [21]. In this setting, it has been proposed that the source of circulating DNA may be from either cellular necrosis or apoptosis [22]. The amount of circulating tumour cells required to generate the levels of DNA detected does not always correspond, however, leading to speculation that DNA may be actively secreted by tumour cells and potentially by other cells under stress [23]. Indeed, in the setting of sepsis, apart from necrosis and apoptosis, the sepsis is associated with many other forms of cellular stress [14], allowing for active secretion as a potential third mechanism in this scenario.

Interestingly, in our study no correlation was observed between plasma DNA concentrations and the three biochemical markers already used to assess prognosis (C-reactive protein, brain natriuretic peptide and procalcitonin). This would indicate that the mechanisms necessary for release of DNA are different to those implicated in the release of these three biochemical markers. Apart from apoptosis, mechanisms such as ischaemia-reperfusion, haemolysis and complement-related pathways may contribute to DNA release. In the case of apoptosis, Zeerleder and colleagues have demonstrated that nucleosomal-related DNA levels were significantly correlated with IL-6, IL-8, elastase-a_1_-antitrypsin complexes and plasminogen activator inhibitor type 1 [16]. Clearly, the source of free plasma DNA in the ICU setting is complicated by the coexistence of several potential mechanisms of DNA release. Much more work is needed in this area.

The clearance mechanisms of plasma DNA have yet to be elucidated. Previous studies have demonstrated that patients with cancer excrete free DNA in the urine equivalent in concentration to that in the plasma, suggesting that the kidneys are an important mechanism of clearance [7]. Furthermore, patients with high levels of circulating DNA were more likely to require at least 24 hours of renal replacement therapy. High levels in this situation, however, may be due to poor renal clearance or to increased cellular damage/secretion, leading to the question of whether circulating DNA is a 'marker or a mediator'.

We recognise that methodological limitations exist in this study. Comparison of results from different papers has been made more difficult due to the variety of techniques used. The inconsistency between publications has been widely debated, but it has been well documented that the preanalytical preparation of cell-free DNA samples is crucial [24] and highlights the necessity for standardisation of sample preparation if findings across studies are to be comparable. Another inconsistency is the units used to describe plasma DNA measurement (genome equivalents/l versus ng/ml) [11,21,22]. One genome equivalent is the amount of DNA present in one cell and is approximately equal to 6.6 pg. In the present study, using the Roche Lightcycler PCR technology, presentation of results takes about 3 hours, making the test a potentially realistic and useful one in the clinical setting (when predicting outcome early in the course of a disease process). Finally, we used the SOFA score as a comparator with plasma DNA levels in our population group. It would be of great interest to use other scoring systems of illness severity such as the Simplified Acute Physiology Score II or the Acute Pathophysiology, Age and Chronic Health Evaluation II in future studies.

## Conclusion

This study has revealed the potential use of plasma DNA in predicting the prognosis of ICU patients as well as the development of sepsis. In fact, the plasma DNA concentration in our patients was shown to be superior to the SOFA scoring system in predicting ICU mortality and could predict inhospital mortality at 3 months.

## Key messages

• 	Free circulating plasma DNA levels are raised in ICU patients compared with those in controls.

• 	Free circulating plasma DNA levels are higher in ICU and hospital nonsurvivors compared with those in survivors.

• 	Free circulating plasma DNA levels are higher in patients with sepsis compared with those in nonseptic patients.

• 	Free circulating plasma DNA levels are superior to the SOFA score at predicting hospital mortality.

• 	Free circulating DNA may be a useful prognostic marker in the ICU setting.

## Abbreviations

ICU = intensive care unit; IL = interleukin; PCR = polymerase chain reaction; ROC = receiver operator curve; SOFA = Sepsis-related Organ Failure Assessment.

## Competing interests

The authors declare that they have no competing interests.

## Authors' contributions

AR, HT and SJW analysed the data, performed statistical analyses and drafted the manuscript. HT performed the molecular analyses, collected the data and helped draft the manuscript. AR, HT, PC and EB conceived of the study, participated in its design and helped draft the manuscript. All authors read and approved the final manuscript.

## Figures and Tables

**Table 1:** Baseline characteristics of patients entered into the study

*n*	52
Age (years) (mean ± standard deviation)	62 ± 18
Male:female sex ratio	32:20
Reason for admission	
Operative	33
Elective vascular surgery	18
Vascular	5
Gastrointestinal	6
Orthopaedic	3
Urology	3
Other	1
Emergency surgery	15
Laparotomy	10
Trauma	2
Other	3
Non-operative	19
Respiratory	8
Cardiac	4
Other	7
Sepsis-related Organ Failure Assessment score on admission	5.5 (3–8)
Admission clinical characteristics	
Sepsis	19
Renal failure	8
Inotropic therapy	16
Mechanical ventilation	36

**Table 2:** Median plasma DNA levels for control samples, presented depending on whether the patients survived intensive care, survived hospital or did not survive

	Plasma DNA (ng/ml)		*P *value
		
	Survivors	Nonsurvivors	
Control	17 (14–19)		
Intensive care	71 (46–113)	321 (185–430)	<0.001
Hospital	68 (47–103)	260 (151–380)	0.001

Data presented as median with interquartile range.

**Table 3:** Univariate analysis: comparison of factors predicting intensive care mortality

	Survived (*n *= 39)	Died (*n *= 13)	*P *value
Age (years)			
Median (interquartile range)	70 (19)	74 (11)	0.49
Minimum–maximum	16–85	36–81	
Male sex [*n *(%)]	14 (36)	6 (46)	0.39
Operative reason for admission [*n *(%)]	26 (67)	7 (54)	0.38
Admission Sepsis-related Organ Failure Assessment score			
Median (interquartile range)	4 (5)	8 (4)	0.004
Minimum–maximum	0–15	2–13	
Organ failures within the first 24 hours			
Cardiovascular failure [*n *(%)]	11 (28)	7 (54)	0.11
Renal failure [*n *(%)]	4 (10)	4 (31)	0.09
Respiratory failure [*n *(%)]	25 (64)	11 (85)	0.30
Admission with sepsis [*n *(%)]	12 (31)	7 (54)	0.19
Co-morbidities of patients prior to admission			
Chronic lung disease [*n *(%)]	6 (15)	1 (8)	0.66
Ischaemic heart disease [*n *(%)]	6 (15)	5 (42)	0.11
Diabetes mellitus [*n *(%)]	5 (13)	2 (17)	0.99
Chronic renal failure [*n *(%)]	1 (3)	1 (8)	0.44
Chronic liver failure [*n *(%)]	0 (0)	1 (8)	0.25
Congestive cardiac failure [*n *(%)]	3 (8)	1 (8)	0.99
Hypertension [*n *(%)]	18 (46)	5 (42)	0.75
Plasma free DNA level (ng/ml)			
Median (interquartile range)	71 (67)	321 (189)	<0.001
Minimum–maximum	8–1,622	40–8,849	
